# At-School Telerehabilitation for Rett Syndrome: Support Teachers Driving Cognitive and Communication Progress in a Randomized Trial

**DOI:** 10.3390/children12070928

**Published:** 2025-07-14

**Authors:** Rosa Angela Fabio, Samantha Giannatiempo, Michela Perina

**Affiliations:** 1 Department of Biomedical, Dental and Morphological and Functional Imaging Sciences, University of Messina, 98123 Messina, Italy; 2 Airett Centre for Research and Innovation, 37100 Verona, Italy; samantha.giannatiempo@centrotice.it (S.G.); michela.perina@airett.it (M.P.)

**Keywords:** Rett syndrome, telerehabilitation, cognitive prerequisites, communication, GAIRS scale, remote intervention, neurodevelopmental disorders

## Abstract

**Background/Objectives:** This exploratory study examined the potential effectiveness of cognitive enhancement interventions targeting basic cognitive prerequisites and communicative abilities in girls with Rett syndrome. Special attention was given to evaluating telerehabilitation as a feasible alternative to traditional in-person therapy, particularly for individuals with severe impairments and limited access to care. **Methods:** Twenty-four girls diagnosed with Rett syndrome (mean age = 13.7 years, SD = 7.1), all meeting the basic cognitive prerequisites defined by the GAIRS scale, were randomly assigned to two groups: a telerehabilitation group (n = 12) and an in-person rehabilitation group (n = 12). Interventions were delivered in school settings and focused on two core areas: basic cognitive skills (e.g., object recognition, spatial and temporal concepts, form and color discrimination, and cause–effect reasoning) and communication skills (e.g., comprehension and expression through gestures, images, or verbal output). **Results:** Both groups showed significant improvements in the cognitive and communicative domains, with generally comparable outcomes. Notably, the telerehabilitation group demonstrated relatively greater gains in verbal expression and cause–effect understanding. Correlational analyses indicated positive associations between the cognitive and communicative improvements, particularly between spatial understanding and expressive abilities. However, these findings should be interpreted with caution due to the sample size and study design limitations. **Conclusions:** These preliminary findings suggest that cognitive enhancement programs may support developmental gains in girls with Rett syndrome and that telerehabilitation could represent a viable alternative for those unable to access in-person care. Given the limited sample size and absence of qualitative measures, further research is necessary to validate its effectiveness and understand its role within comprehensive care models.

## 1. Introduction

### 1.1. Rett Syndrome

Rett Syndrome (RTT) is a rare genetic neurodevelopmental disorder that primarily affects females, with an estimated prevalence of approximately 1 in every 10,000 female births [1,2]. The condition is most commonly linked to mutations in the MECP2 gene, which plays a critical role in neural development and synaptic function [2]. RTT typically follows a period of apparently typical early development, after which individuals may experience a phase of functional regression, particularly affecting their motor and communication domains. The key clinical features include changes in hand function, with reduced purposeful use and the emergence of repetitive movements (such as wringing or tapping), along with significant challenges in verbal communication and global motor coordination, often framed in terms of apraxia or dyspraxia [3,4].

In addition to its core neurological aspects, RTT is frequently accompanied by a constellation of complex comorbidities. These may include scoliosis and other orthopedic issues, breathing irregularities (such as breath-holding and hyperventilation) [5], significant gastrointestinal disturbances (including chronic constipation and reflux), and dysautonomia [6]. Medications may target seizures, sleep problems, autonomic dysfunction, or behavioral symptoms, offering partial relief and stabilization [7]. However, rehabilitation remains the cornerstone of care, aiming to preserve functional abilities, enhance engagement, and support the overall well-being of both individuals and their families.

Given the progressive nature of RTT, individuals require lifelong, continuous, and personalized rehabilitation. Evidence indicates that high-frequency, low-intensity therapeutic interventions can lead to meaningful improvements in both motor and cognitive domains [7]. Fabio et al. [8] emphasize that tailoring interventions to the individual’s specific needs and abilities maximizes both functional gains and quality of life. The study highlights how early, personalized approaches that adapt over time are critical for supporting development in RTT patients. Moreover, the active involvement of families and caregivers is essential in reinforcing therapy goals and ensuring continuity outside of clinical settings.

Nevertheless, the complex cognitive and communicative profiles often associated with RTT can pose significant challenges to the effectiveness of conventional interventions. These challenges, together with the diverse motor planning profiles observed in individuals with RTT, highlight the critical need for innovative, individualized approaches that prioritize cognitive stimulation and support the development of expressive and receptive communication abilities, respecting the varied neurodevelopmental trajectories that are characteristic of this population.

Recent studies have highlighted the potential of telerehabilitation (TR) as an effective modality for delivering therapy remotely [9,10]. For instance, Caprì et al. [11] introduced a structured TR model for individuals with RTT, emphasizing the feasibility of cognitive and motor interventions administered virtually. The integration of digital tools and virtual environments may further enhance the accessibility, personalization, and engagement of therapy, particularly important for individuals with limited mobility or those living in areas with reduced access to specialized services [12,13].

Furthermore, literature suggests that digital rehabilitation programs that are individualized, goal-directed, and embedded within daily routines across home and school settings tend to yield more sustainable developmental benefits [14,15,16].

However, despite its advantages, telerehabilitation may also pose significant challenges. These include technological barriers such as limited internet connectivity, inadequate access to communication devices, and difficulty in building effective patient–therapist relationships remotely. Such issues have been documented in the broader literature on TR, highlighting the importance of appropriate implementation strategies to mitigate these limitations [17].

### 1.2. Inclusive Education in Italy

Since school represents a key environment for the implementation of such interventions, particularly in pediatric populations, it is crucial to consider how educational systems integrate rehabilitation strategies.

Inclusive education in Italy guarantees access to public schooling for all children with disabilities, including those with RTT. Each child is supported by an individual special education teacher, whose role is to facilitate the acquisition of motor, cognitive, communicative, and social skills. However, these professionals are often not specifically trained to address the complex needs associated with rare neurodevelopmental conditions such as RTT.

In many inclusive classrooms, educational activities tend to prioritize motor skills and personal autonomy, while structured cognitive stimulation remains largely overlooked. Beyond this imbalance, school environments may inadvertently contribute to formal inclusion without substantive participation by adopting generalized approaches that fail to meaningfully engage students with RTT or to tailor learning experiences to their specific cognitive profiles. This lack of personalization risks marginalizing their intellectual potential and limiting opportunities for participation in cognition-enriching activities.

This dynamic exemplifies what scholars have termed symbolic exclusion—a condition in which students are physically present in inclusive classrooms but remain cognitively or socially disengaged due to the absence of tailored support. In such contexts, inclusion becomes procedural rather than substantive, with participation reduced to mere access rather than meaningful engagement. Without individualized strategies, school environments risk reinforcing inequalities under the guise of formal equity. This results in significant gaps in theoretical knowledge and cognitive development among girls with RTT.

Such challenges are not exclusive to RTT. Children with other chronic conditions, often face similar marginalization in inclusive settings due to the insufficient training of school personnel and the absence of individualized educational responses. As highlighted by Kucharska [18], the lack of adequate systemic preparation leads to forms of exclusion and social isolation, even in contexts formally committed to inclusion. As Grigorenko et al. [19] emphasize, effective inclusion must go beyond access and ensure personalized, evidence-based interventions that are cognitively oriented and responsive to the specific profiles of students with neurodevelopmental and learning disorders. Moreover, involving families in the educational process can strengthen collaboration, promoting synergy between teachers and caregivers to reinforce the strategies applied in school at home. Families and professionals play a crucial role in representing the needs and experiences of individuals, ensuring that interventions are both meaningful and tailored. Without such individualized support, general education environments often rely on a “one size fits all” instructional model, which fails to address the heterogeneity of learning needs and contributes to persistent academic and social inequalities. In our approach, the teachers maintained regular communication with the parents, who were encouraged to support and generalize the cognitive and communicative activities at home.

Furthermore, there is an urgent need to provide special education teachers with targeted training that enables them to recognize and respond to the complex and often subtle manifestations of conditions such as RTT [20]. As shown in the Polish model discussed by Kucharska [18], the introduction of specialized figures such as health educators—professionals with specific knowledge of chronic conditions and their impact on learning—can significantly improve educational support for students with disabilities. Adopting similar specialized roles or enhancing the training of existing special education teachers may represent a concrete step toward bridging the gap between formal inclusion and actual participation.

Despite the limitations of the current inclusive model, in Italy each girl with RTT is typically assigned a dedicated team, including a special education teacher and an educator, who work together to adapt classroom activities to the child’s needs. However, cognitive-specific interventions remain limited in these inclusive settings. This gap highlights the need for complementary approaches. In this context, telerehabilitation (TR) emerges as a promising tool to deliver personalized cognitive support in accessible and sustainable ways. However, while TR offers a promising avenue for delivering targeted interventions in inclusive school contexts, several barriers must be considered. These include technological limitations in under-resourced schools, variability in the digital literacy and engagement of teachers, and the need for ongoing supervision to ensure the fidelity of implementation. Recognizing these challenges is essential to develop realistic and sustainable intervention models.

### 1.3. Telerehabilitation as a Tool for Cognitive Rehabilitation

Growing evidence supports the feasibility and clinical potential of cognitive telerehabilitation (TR), which is defined as the remote delivery of structured, therapist-guided cognitive training sessions using digital platforms. Positive outcomes have been reported in children with complex neurodevelopmental disorders [21], and meta-analyses show significant improvements in attention, executive function, and global cognition in individuals with acquired brain injuries and other neurological conditions [22,23]. A recent expert consensus confirmed that TR is safe, well-tolerated, and effective across domains such as attention, memory, executive functioning, and language in conditions like stroke, traumatic brain injury, multiple sclerosis, and dementia [24]. Nevertheless, challenges such as protocol heterogeneity, small sample sizes, a lack of standardized outcome measures, and technological literacy issues persist. Additional studies support TR as a valid model of care [25,26,27], particularly when caregivers are involved and adaptive, engaging platforms are used.

In the context of developmental learning disorders, Casalini and Pecini [28] further demonstrated that TR can effectively support cognitive training in children with dyslexia, offering individualized, accessible, and engaging interventions with outcomes comparable to traditional in-person therapy.

Within RTT specifically, Iannizzotto et al. [29] implemented a telerehabilitation protocol focused on motor function and reported improvements in upper limb mobility through computer vision–based systems. However, despite clear and pressing needs, structured cognitive programs specifically tailored to the RTT profile remain scarce in both research and practice

In response to these needs, a six-month school-based cognitive enhancement program was delivered via telerehabilitation. This intervention aimed to complement inclusive education with structured, individualized cognitive activities within the school day, supported by adaptive technologies and pedagogical strategies specifically designed for this population. It also aimed to empower special education teachers through hands-on implementation and guided support.

### 1.4. Study Hypotheses

**H1.**  *We hypothesize that both the experimental group (remote support) and the control group (in-person cognitive rehabilitation) will show improvements in both basic cognitive abilities and communicative skills at the post-intervention time point.*

This expectation is based on existing evidence suggesting that individuals with Rett syndrome can benefit from structured and repeated stimulation targeting foundational skills, especially when support is mediated through stable relational figures such as special education teachers. Additionally, both telerehabilitation and in-person interventions have been shown to be feasible and potentially effective in individuals with complex neurodevelopmental conditions.

**H2.**  *We hypothesize that basic cognitive abilities and early communicative skills will exhibit a significant association. This hypothesis is informed by developmental theories positing that cognitive and communicative domains are interdependent, rather than hierarchically or casually ordered. Foundational cognitive processes—such as object permanence, causal reasoning, and sequential processing—are thought to facilitate the emergence of intentional communicative behaviors by enabling the infant to interpret and anticipate agent–object and agent–agent interactions. For instance, recognizing the functional properties of objects or predicting action outcomes may support the development of communicative strategies such as gestural requests or symbolic referencing. Nonetheless, we recognize that this relationship is not mechanistic: it is modulated by a constellation of contextual and individual factors, including social engagement, communicative input, and motivational drivers, which collectively influence developmental outcomes. Therefore, any observed association should be interpreted as probabilistic rather than deterministic, reflecting the influence of environmental supports and interactional opportunities on the extent to which cognitive gains translate into communicative improvements.*

## 2. Materials and Methods

### 2.1. Participants

A total of 24 girls and adolescents diagnosed with RTT were enrolled in the study. Prior to participation, each child’s legal guardian was given a detailed document outlining the nature and objectives of the study and subsequently provided written informed consent.

Inclusion criteria were as follows: (1) confirmed diagnosis of RTT; (2) regular attendance in a school setting; and (3) access to basic digital tools (e.g., tablet or computer with internet connectivity) to enable participation in remote sessions. Eligibility also requires that participants meet the basic cognitive prerequisites as defined by the Global Assessment and Intervention Rating Scale (GAIRS) [30], ensuring their ability to engage meaningfully in the proposed activities. Specifically, they had to achieve a score of at least 4 out of 5, indicating correct and consistent performance across three separate sessions.

Participants were then randomly assigned to one of two groups: an experimental group (n = 12), which received an at-school structured six-month cognitive telerehabilitation program, and a control group (n = 12), which continued to receive standard educational support within their school environment, without additional cognitive intervention. It should be noted that assessors were not blinded to participants’ group allocation, due to the practical impossibility of concealing diagnostic or behavioral features that were often readily observable, all evaluations were conducted using standardized administration procedures. To reduce potential bias, a subset of sessions was independently reviewed and scored by a second trained rater, and inter-rater agreement was monitored to ensure scoring reliability.

At baseline, the two groups were comparable in terms of age, clinical profile, and functional levels as seen in Table 1.

### 2.2. Study Design

This study followed a longitudinal, controlled experimental design composed of four phases: a baseline assessment (T0), a three-month midpoint evaluation (T1), a post-intervention evaluation after six months of treatment (T2), and a follow-up phase conducted three months after the intervention ended (T3). The follow-up phase corresponded to the summer school break and included no structured educational input.

After recruitment and informed consent, participants were randomly assigned to one of two groups: an experimental group, which received a structured cognitive telerehabilitation program, or a control group, which followed standard in-person educational activities.

The standardized tools used during baseline assessment included the Rett Assessment Rating Scale (RARS) [31] to evaluate syndrome severity and the Vineland Adaptive Behavior Scales (VABSs) [32] to assess adaptive functioning. All assessments were conducted by certified therapists trained in Rett Syndrome, either in person or remotely.

The primary measure was the Global Assessment and Intervention Rating Scale (GAIRS) [31], a multidimensional checklist tailored for individuals with Rett Syndrome and complex neurodevelopmental profiles. Only two GAIRS subscales (i.e., Basic Cognitive Concepts and Communication Abilities) were analyzed in this study.

### 2.3. Assessment and Measures

Prior to the intervention, information about the participants’ characteristics was gathered using the RARS and VABSs.

RARS is a validated clinical tool used to evaluate the severity of RTT symptoms across seven functional domains: cognitive, sensory, motor, emotional, autonomy, typical characteristics, and behavior. The scale includes 31 items rated on a 4-point Likert scale (1 = within normal limits; 4 = marked abnormality), allowing classification of severity into mild (0–55), moderate (56–81), or severe (>81). The instrument demonstrates high psychometric properties, including high internal consistency (Cronbach’s alpha = 0.912).

The VABSs assess four core domains of adaptive functioning: communication, daily living skills, socialization, and motor abilities. Each item is rated by an interviewer (2 = always, 1 = sometimes, 0 = rarely/never), and domain scores are summed to yield an overall adaptive behavior composite. VABSs have well-established reliability indices across domains, with split-half reliability ranging from 0.70 to 0.95 and interrater reliability from 0.62 to 0.75.

The GAIRS Checklist was administered at four time points (T0, T1, T2, and T3) and served as the primary outcome measure for evaluating participants’ cognitive and communicative functioning over time.

GAIRS encompasses 10 functional domains: basic prerequisites, neuropsychological skills, basic cognitive concepts, advanced cognitive concepts, communication, emotional-affective abilities, hand motor skills, graphomotor abilities, gross motor abilities, and autonomy in daily life. In this study, the intervention specifically targeted two subscales: the Basic Cognitive Concepts area (e.g., object recognition, form and color discrimination, spatial and temporal understanding, and cause–effect relationships) and the Communication Abilities area (e.g., comprehension and expression through gestures, images, and verbal output). The items included in the targeted subscales are briefly described in Table 2, while the evaluation procedures and examples of the materials used during the assessment are provided in Appendix A and Appendix B. Recent studies have demonstrated that GAIRS is a reliable and comprehensive instrument for evaluating individuals with Rett syndrome in both traditional and remote settings [8].

### 2.4. Procedure

After initial recruitment and consent, all participants underwent a baseline assessment to determine eligibility and establish initial functional profiles. This included administration of the Rett Assessment Rating Scale (RARS) to evaluate syndrome severity, the Vineland Adaptive Behavior Scales (VABSs) to assess adaptive functioning, and the Global Assessment and Intervention Rating Scale (GAIRS) to capture cognitive and communicative abilities across multiple domains.

Participants were then randomly assigned to the experimental or control group. The experimental group received a six-month cognitive and communication telerehabilitation program, conducted at school by their special education teacher in collaboration with a remote therapist. The intervention targeted selected items from the GAIRS subscales for Basic Cognitive Concepts and Communication Abilities.

**Figure 1:**
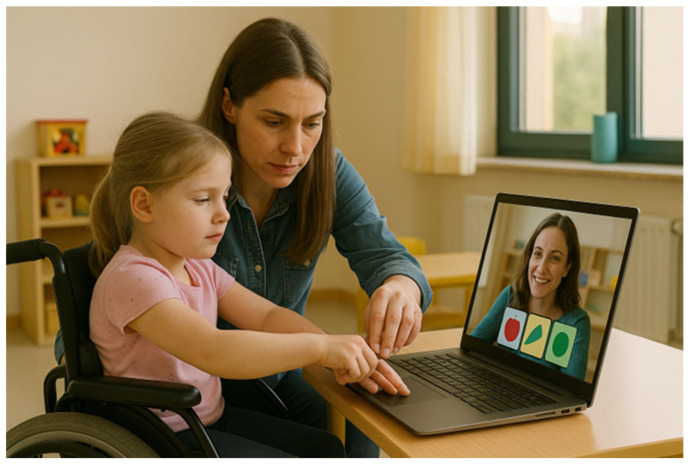
The therapist and the teacher guide the child through a discrimination task as part of the cognitive rehabilitation program conducted at school.

To ensure engagement and maximize educational collaboration, the intervention consisted of at least three sessions per week, each lasting approximately one hour, although session duration was adapted to each participant’s attention span. Sessions were conducted during school hours, in quiet, structured environments within the school setting. Figure 1 shows the therapist, the teacher, and the child engaged in a discrimination task.

When possible, activities included peer involvement, encouraging participation in simple shared tasks to support communication and social interaction.

Interventions were delivered remotely through a simple, user-friendly video call platform, allowing real-time interaction between therapists, teachers, and girls. All necessary cognitive enhancement materials and communication tools were provided to the school and adapted to the developmental level and functional profile of each participant. These included visual support, representing symbolic communication systems designed to support attention, comprehension, and expressive abilities.

Each session followed a consistent structure: (1) warm-up activities to engage attention (e.g., songs and visual cues); (2) core cognitive tasks targeting basic processes such as object permanence, categorization, sequencing, and visual discrimination; and (3) communicative activities involving gesture imitation, symbol matching, and cause–effect games using adapted digital tools. The intervention was highly individualized, with activities tailored to each student’s cognitive profile, and materials adapted to accommodate motor and sensory limitations. To guarantee fidelity of implementation, teachers were instructed to record each intervention session. Additionally, periodic supervisions were conducted by the research team to review session recordings and provide feedback, ensuring adherence to the intervention protocol across all settings. Family members were encouraged to generalize activities at home when possible, supporting continuity across settings.

The special education teachers involved in the intervention completed an online training course provided by the Italian Rett Syndrome Association (AIRETT). This comprehensive training covers all key domains of Rett Syndrome, including cognitive, communicative, motor, speech therapy, and occupational aspects, and offers both theoretical knowledge and practical advice on approaches and strategies to effectively support individuals with this condition. Further details on the course can be found on the AIRETT website (https://www.airett.it/presentazione-corso-online-2024-2025, accessed on 20 June 2025).

Participants were evaluated at the following time points:

- T0 (Baseline): Prior to intervention.
- T1 (Midpoint): After three months of training.
- T2 (Post-intervention): After six months of training.
- T3 (Follow-up): Three months after the end of the intervention, during a period with no rehabilitation activity (i.e., the summer school break).

At each point, the previously mentioned subscales of the GAIRS Checklist were administered as the primary outcome measure to monitor changes in cognitive and communicative functioning.

To minimize variability between the face-to-face and remote delivery modalities, both groups followed the same structured protocol in terms of frequency (minimum three sessions per week), duration (approximately one hour), and targeted skills. The intervention content, materials, and therapeutic goals were standardized and aligned with the GAIRS subscale items. All participants received individualized materials adapted to their developmental level, regardless of group allocation. Special education teachers in both conditions completed the same certified training course and received detailed implementation guidelines. Additionally, the remote therapist maintained close communication with onsite staff to ensure consistency in delivery, troubleshoot challenges, and monitor fidelity to the intervention model.

To assess the overall satisfaction and acceptability of the telerehabilitation service, a post-intervention questionnaire was administered to both the parents of the children involved and the therapists (teachers/educators) who delivered the activities. Parents were asked to evaluate their general satisfaction with the service, their perceived level of involvement in the project, and their global assessment of the quality, usefulness, and accessibility of the intervention experience. Responses were collected using a 5-point Likert scale (ranging from 1 = very dissatisfied to 5 = very satisfied), along with open-ended questions aimed at gathering qualitative feedback. Therapists were invited to complete a more detailed questionnaire, which focused on the ease of implementation of the intervention protocol, the usability of technological tools, the quality of communication with families and team members, and their perceived effectiveness of the intervention. The same 5-point Likert scale was used, complemented by optional narrative sections for further qualitative insights. While these narrative responses were not analyzed using formal qualitative methods, they were used to informally identify common themes and to support interpretation of the quantitative results.

### 2.5. Statistical Analysis

Statistical analyses were performed using IBM SPSS Statistics (Version 24; IBM Corp., Armonk, NY, USA).

Data from the two subscales of the GAIRS Checklist were analyzed following standardized procedures [30] and an average score was calculated for each subscale. Scores ranged from 1 to 5, with higher scores indicating greater performance within the corresponding functional domain.

The normality of the data distribution was evaluated using both the Shapiro–Wilk test and visual inspection. No significant deviations from normality were observed at any of the three assessment points (T0, T1, and T2), with all Shapiro–Wilk *p*-values exceeding 0.05 and W statistics ranging from 0.92 to 0.98.

As the assumption of normality was met, two separate within-subjects ANOVAs were performed to assess the impact of telerehabilitation over time and individual differences in performance. The first analysis was a 2 (groups: Experimental vs. Control) × 14 (basic cognitive abilities) × 4 (time points: baseline, post-test 1, post-test 2, and follow-up) ANOVA focusing on basic cognitive skills. The second involved a 2 (groups: Experimental vs. Control) × 9 (communication abilities) × 4 (time points: baseline, post-test 1, post-test 2, and follow-up) ANOVA, also within-subjects, examining communication abilities.

When significant effects emerged, effect sizes were calculated to quantify their magnitude. For the ANOVA results, eta squared (η^2^) was used and categorized according to established guidelines [33]. Additionally, paired-sample *t*-tests comparing performance across different time points were accompanied by Cohen’s d effect sizes, interpreted as small (0.2), medium (0.5), or large (0.8) changes. These effect sizes provided further insight by illustrating the extent of change between specific measurement occasions, complementing the overall ANOVA findings. To control for Type I error due to multiple comparisons, Bonferroni correction was applied where appropriate.

## 3. Results

### 3.1. Basic Cognitive Abilities

Table 3 reports mean scores and standard deviations across time points. A within-subjects ANOVA revealed a significant main effect of time on all basic cognitive abilities, *p* < 0.05, with the partial eta squared (η^2^ₚ) values ranging from 0.06 to 0.15.

**Figure 2:**
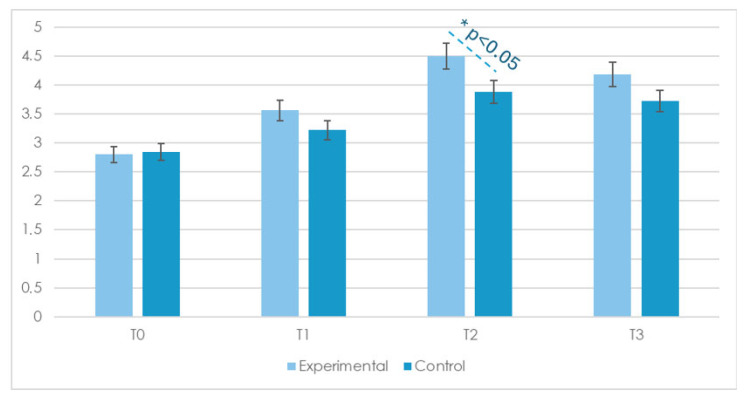
Compound mean scores on the Basic Cognitive Skills items at four time points (T0 = baseline, T1 = 3 months, T2 = 6 months, and T3 = 9-month follow-up) for the Experimental and Control groups; * = *p* < 0.05 for between-group comparison (independent sample *t*-test). Only statistically significant differences are marked. T-values, *p*-values, effect sizes (Cohen’s *d*), and 95% confidence intervals for comparisons between T0 and T2 are reported in Appendix C.

These results suggest that the interventions were effective in improving participants’ adaptive cognitive functioning over time. No group by time and no group by basic cognitive abilities interactions were found. Post hoc comparisons indicated significant improvements from T0 to T1 and from T0 to T2 for most abilities (*p* < 0.01), whereas differences between T1 and T2 were generally smaller and did not reach statistical significance. No significant changes were observed in the following abilities: use of context-based vocalizations and participation in need-based exchanges. Further paired sample *t*-tests confirmed these findings. As can be seen in Figure 2, which presents the compound score (i.e., the mean of all items from the Basic Cognitive Skills section), significant improvements were observed between T0 and T1 as well as between T0 and T2 for several abilities, with t(23) values ranging from 1.81 to 4.11, *p* < 0.05, and the corresponding Cohen’s d values ranging from 0.26 (small to moderate) to 0.81 (large). The changes between T1 and T2 and between T2 and T3 were generally smaller and non-significant, suggesting that most improvements occurred during the initial phase of the intervention and that the acquired skills were maintained at follow-up (T3), after the summer break. Although no significant Group × Time interactions were found, follow-up independent sample *t*-tests revealed significant between-group differences for Object Recognition (T2 and T2) and Spatial Concepts (T2 and T3), in favor of the experimental group. These differences are indicated in Table 3 and Figure 2 with asterisks. While the results were statistically significant before correction (*p* < 0.05), they did not remain so after applying the Bonferroni adjustment (corrected *p* = 0.18), indicating a trend rather than a robust effect.

These improvements are likely to translate into enhanced everyday functioning and increased participation for individuals with Rett syndrome. Given the complexity of the condition, even moderate cognitive gains can significantly impact autonomy in daily activities such as problem-solving during routine tasks, improved anticipation of events, and greater ability to engage in social interactions. For example, improvements in cause–effect reasoning and object recognition may facilitate more effective communication attempts and adaptive responses, thereby enhancing meaningful participation in both educational and social settings. Moreover, the maintenance of these skills at follow-up suggests sustained benefits from the intervention, reinforcing its practical value in supporting not only cognitive development but also improved engagement and participation in daily life, thereby contributing positively to overall quality of life.

### 3.2. Communication Abilities

**Figure 3:**
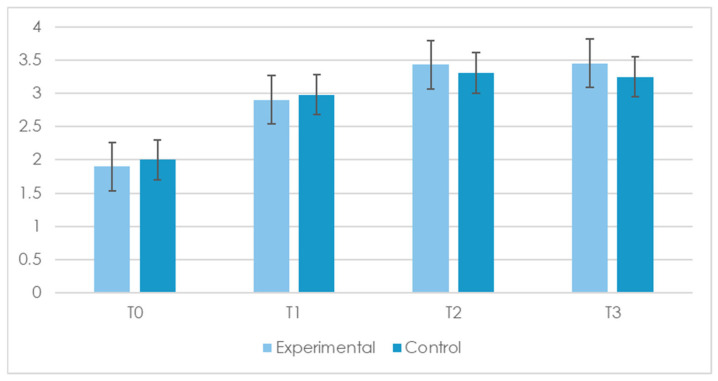
Compound mean scores on the Communication Skills items at four time points (T0 = baseline, T1 = 3 months, T2 = 6 months, and T3 = 9-month follow-up) for the Experimental and Control groups. T-values, *p*-values, effect sizes (Cohen’s *d*), and 95% confidence intervals for comparisons between T0 and T2 are reported in Appendix D.

With reference to communication abilities, only three participants demonstrated the ability to complete the tasks involving written communication. Because this number was insufficient for a meaningful group-level statistical analysis, Items 10 and 11, recognizing and expressing a basic need through the written word and understanding the correspondence between a picture and the written word, were specifically excluded from the statistical analyses. However, all participants remained included in the overall sample for all other analyses (Table 4, Figure 3). This exclusion can represent a limitation in the current study, as it reduces the comprehensiveness of the communicative outcome assessment.

A within-subjects ANOVA revealed a significant main effect of time across all remaining communication abilities, *p* < 0.05, with η^2^ₚ values ranging from 0.06 to 0.15. These findings indicate a general improvement throughout the rehabilitation period. No group by time and no group by basic cognitive abilities interactions were found. As can be seen in Figure 3, which presents the compound score (i.e., the means of all items from the Communication section), significant improvements were observed between T0 and T1 and from T0 to T2 for most items (*p* < 0.01), while changes between T1 and T2 were typically smaller and did not reach statistical significance. No significant differences were found for geometric form discrimination, understanding of cause–effect relationships, anticipation of familiar events, human body discrimination, and time concepts.

Complementary paired-sample *t*-tests supported these trends. Significant and substantial improvements were observed from T0 to T1 and from T0 to T2, with t(23) values ranging from 1.81 to 4.11, *p* < 0.01, and effect sizes (d) ranging from 0.26 to 0.81. As with cognitive abilities, improvements appeared to plateau after T2, and the acquired communication skills were largely maintained at follow-up (T3).

Overall, the results demonstrate that both types of rehabilitation can be a powerful tool for enhancing communication and cognitive abilities in girls with RTT. The improvements observed across multiple adaptive domains highlight the potential of remote interventions in fostering cognitive, social, and motor development, paving the way for further research on personalized, technology-assisted rehabilitation approaches.

### 3.3. Correlation Between Cognitive and Communication Skills

**Figure 4:**
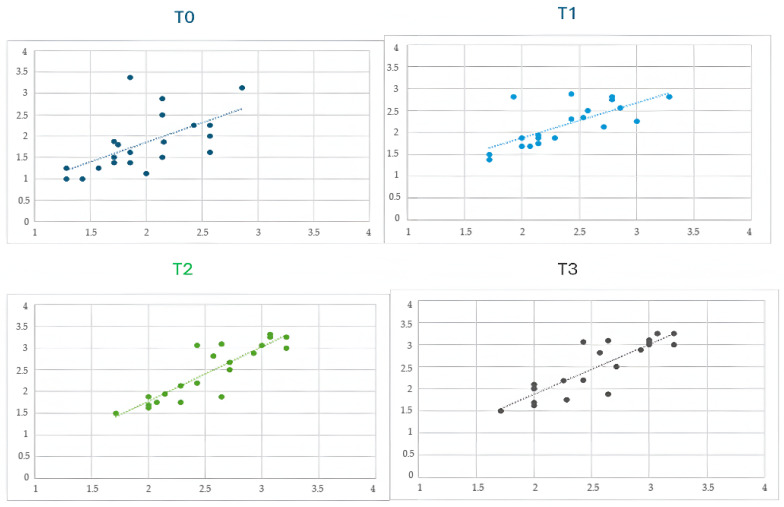
Scatterplots with fitted regression lines showing the correlation between communication scores and basic cognitive skill scores at four time points (T0 = baseline, T1 = 3 months, T2 = 6 months, and T3 = 9-month follow-up). Each point represents an individual participant. The dotted lines indicate linear regression fits for each time point. Pearson correlation coefficients were as follows: T1, r = 0.69, *p* < 0.01; T2, r = 0.67, *p* < 0.01; T3, r = 0.73, *p* < 0.01; and T4, r = 0.79, *p* < 0.01.

To examine the relationship between cognitive and communicative development, Pearson correlation coefficients were calculated between the composite scores (i.e., summed total scores) of cognitive abilities and communication abilities at each time point. The results indicated consistently strong associations across all time points, with correlation coefficients ranging from r = 0.69, *p* < 0.01 at time T1; r = 0.67, *p* < 0.01 at T2; r = 0.73, *p* < 0.01 at T3; and r = 0.79, *p* < 0.01 at T3. These findings suggest that gains in cognitive functioning were closely linked to concurrent improvements in communicative competencies (Figure 4). However, these correlations do not imply a direct causal relationship. It is possible that unmeasured contextual or interpersonal variables—such as family engagement, teacher motivation, or classroom climate—may have contributed to improvements in both domains, influencing the strength of the observed associations.

Finally, we assessed the overall satisfaction and acceptability of the telerehabilitation service for both parents and teachers. The results showed a high level of satisfaction from both groups. Most parents (92%) reported high satisfaction scores (4 or 5), and 89% felt actively involved throughout the project. Additionally, over 85% provided a positive overall evaluation of the intervention, frequently highlighting the accessibility of the service and the opportunity it provided to better monitor their child’s progress. The therapists also reported a positive experience: the majority found the protocol easy to implement (83%), and 90% rated their communication with families and colleagues as good or very good. Furthermore, 87% considered the intervention effective in achieving its objectives, and 78% expressed confidence in the long-term sustainability of the telerehabilitation model. The qualitative analysis of the open-ended responses revealed recurring themes, including the flexibility of the intervention framework, the value of home–school collaboration, and the perception that the home setting fostered greater engagement and participation from the children.

## 4. Discussion

The present study aimed to evaluate the effects of a school-based cognitive telerehabilitation program for girls with RTT, comparing remote versus in-person interventions on basic cognitive and communicative abilities. The results partially confirmed our hypotheses, offering valuable insights into the potential and limitations of individualized rehabilitation strategies for this population.

### 4.1. Cognitive and Communicative Improvements

In line with Hypothesis 1 (H1), both groups showed significant improvements in cognitive and communicative functioning, and these gains were maintained during the follow-up phase. These preliminary results suggest that telerehabilitation may offer comparable benefits to in-person intervention [33,34]; specifically, the results indicate that the most substantial gains occurred during the initial phase of the program (T0–T1), with these improvements largely maintained at follow-up (T3), suggesting the long-term retention of acquired skills even after a period of reduced training intensity (i.e., summer break). However, caution is warranted in interpreting these findings given the limited sample size and design constraints. Further research with larger samples and rigorous controls is needed to confirm the equivalence of the two modalities [8,10] The observed effect sizes, ranging from small to large, underscore the clinical relevance of the gains. The absence of significant group-by-time interactions suggests that both delivery modalities (remote and in-person) were equally effective, a finding that aligns with prior research showing the feasibility and comparability of telerehabilitation and traditional face-to-face therapy in neurological populations [23,24]. These outcomes are especially encouraging considering the severe communicative and motor impairments typically associated with RTT, which often hinder the efficacy of conventional interventions.

Notably, although the program incorporated remote therapist involvement, children maintained direct in-person interaction with their special education teachers during sessions, preserving essential face-to-face relational engagement. This hybrid approach combines the benefits of remote professional support with the immediacy of live interaction, which may be particularly important for motivation and emotional connection.

Nonetheless, the reduced direct contact between therapists and children through tele-delivery raises important considerations regarding its impact on emotional engagement and motivation. As highlighted by Franco et al. [17], telerehabilitation can face barriers such as difficulty in building effective patient-therapist relationships remotely or issues like reduced emotional engagement and digital fatigue, which may impact the overall effectiveness and sustainability of the intervention.

Future studies should carefully evaluate these relational aspects to optimize telerehabilitation approaches and ensure they meet the complex needs of individuals with RTT.

### 4.2. The Interdependence of Cognitive and Communication Skills

Consistent with Hypothesis 2 (H2), strong correlations were observed between basic cognitive and communicative abilities at all time points. This finding supports the idea that early cognitive functions—such as cause–effect reasoning, anticipation, and object recognition—form a foundational basis for the development of intentional communication. This aligns with the developmental perspective that sees cognition and communication as interdependent processes. However, it is also important to consider that these associations may be shaped not only by intrinsic developmental links but also by extrinsic environmental and systemic factors. For instance, the structured nature of both the in-person and telerehabilitation programs, as well as the supportive roles of teachers and therapists, may have simultaneously stimulated both cognitive and communicative growth. As noted by Grigorenko et al. [19], interventions aimed at fostering inclusion should not focus solely on observable behaviors but must also address the underlying cognitive prerequisites that enable meaningful engagement in social and communicative contexts. The close coupling of cognitive and communication growth observed in this study suggests that improvements in cognitive functioning may have a cascading effect on both expressive and receptive communication skills. However, its effectiveness likely reflects not only the impact of the training itself but also the broader ecosystem of interactional and contextual support within which it is embedded, including systemic factors such as an inclusive classroom climate, positive peer interactions, and cultural attitudes toward neurodiversity and disability. Recognizing and fostering these elements within educational settings may enhance the effectiveness of cognitive and communicative interventions, supporting more meaningful participation and social integration for children with complex neurodevelopmental needs.

### 4.3. Telerehabilitation as a Tool for Inclusive Education

This study contributes to the growing body of evidence supporting telerehabilitation (TR) as an accessible and effective approach to delivering therapy in school contexts, especially for individuals with rare and complex neurodevelopmental disorders. As highlighted by Caprì et al. [11], technology-based interventions can help overcome traditional barriers related to mobility, geographical location, and limited availability of specialized services. Our findings extend this work by showing that cognitive TR can be successfully integrated into the school day and supported by educators, without reducing treatment effectiveness.

Importantly, the program also aimed to empower special education teachers, who are often undertrained in addressing RTT-specific needs [18,19]. The teachers provided detailed feedback indicating their confidence in implementing the intervention, satisfaction with the usability of the telerehabilitation tools, and positive perception of the overall effectiveness of the protocol. They also indicated strong engagement in the project, reflecting positive acceptance of the intervention. It should be noted that the present study did not include a qualitative component (e.g., in-depth family or teacher testimonials), which limits the richness of contextual interpretation of the results. Future research should incorporate qualitative methods to capture participants’ subjective experiences and strengthen the participatory dimension of intervention development.

The structured integration of cognitive goals into daily routines through adaptive digital tools appears to support progress toward bridging the gap between formal inclusion and meaningful participation. While this represents a promising direction, future work should aim to further validate this outcome by incorporating direct input from the children and facilitating the deeper involvement of families in the design process, in line with participatory research principles.

### 4.4. Limitations and Future Directions

Despite promising results, several limitations must be acknowledged. First, the small sample size and the exclusion of some communication items due to participant inaccessibility (e.g., written tasks) limit the generalizability of the findings. Second, although both delivery methods were effective, the study did not explore participant preference, user engagement metrics, or the long-term effects beyond follow-up. Third, the direct participation of the students in the intervention process was necessarily limited. Given the severity of the motor and communication impairments characteristic of the participants, their active involvement was minimal. As a result, meaningful engagement was primarily mediated through teachers and caregivers. Moreover, while improvements were evident, certain cognitive and communication domains (e.g., use of context-based vocalizations and discrimination of geometric forms) showed limited change, indicating that more targeted interventions may be needed for these specific skills.

Additionally, although the satisfaction levels reported by both teachers and parents were high, the study did not systematically evaluate the emotional and relational dimensions of remote delivery. It remains possible that telerehabilitation, while logistically advantageous, may reduce opportunities for spontaneous interpersonal interaction or increase dependence on teacher engagement and digital stamina. Future studies should incorporate specific measures to assess emotional engagement and consider hybrid models that preserve both flexibility and relational depth.

Finally, given the strong correlations observed between cognitive and communicative domains, future research could explore whether improvements in one domain may lead to gains in the other. Moreover, studies might also investigate the potential added value of integrated interventions targeting both domains simultaneously, compared to uni-domain approaches.

## 5. Conclusions

In conclusion, the findings from this study offer empirical support for the effectiveness of school-based cognitive telerehabilitation in girls with RTT, demonstrating significant and sustained gains in both cognitive and communicative functioning. By combining evidence-based digital tools with educator involvement, telerehabilitation can play a key role in fostering inclusive, personalized, and sustainable rehabilitation models. However, caution is warranted when generalizing these findings beyond the specific population and context studied. It is also important to acknowledge the limitations of telerehabilitation, including challenges related to emotional engagement, digital fatigue, and reliance on teacher motivation. Future research should explore hybrid models that integrate the flexibility of remote delivery with the relational depth and contextual responsiveness of in-person interaction in order to maximize effectiveness across diverse educational and clinical settings. These results encourage further integration of cognitive TR within educational contexts and highlight the need for systematic training of school personnel to ensure the successful implementation of technology-assisted cognitive programs for children with rare neurodevelopmental disorders. Future directions should also prioritize participant well-being by incorporating measures that directly assess motivation, emotional engagement, and overall impact on quality of life. Future directions should also prioritize participant well-being by incorporating measures that directly assess motivation, emotional engagement, and overall impact on quality of life. Moreover, future intervention design should, when possible, be informed by neurodiversity-oriented frameworks and include the perspectives of neurodivergent individuals, promoting self-advocacy or, when direct self-advocacy is not feasible—as in the case of individuals with Rett syndrome—advocacy through caregivers to ensure their needs and preferences are represented (see full dataset in Supplementary Materials).

## Figures and Tables

**Table 1:** Characteristics of participants.

ID	AGE	RARS	VABSs	GAIRS Basic Cognitive Abilities	GAIRS Communication Abilities
Experimental Group					
1	4	75.5	75	1.63	2.57
2	10	75.5	75	2.00	2.57
3	3	46	100	2.25	2.43
4	21	58	84	1.63	1.86
5	24	71	71	1.38	1.71
6	17	69.5	109	1.50	1.71
7	24	64	136	1.50	2.14
8	24	62	91	2.50	2.14
9	13	64	111	1.13	2.00
10	6	65.5	104	1.88	1.71
11	14	72	151	3.13	2.86
12	9	70	77	1.86	2.15
Control Group					
1	12	81.5	85	1.25	1.29
2	4	60.5	128	3.38	1.86
3	23	63.5	74	2.88	2.14
4	11	64.5	84	2.25	2.57
5	7	67.5	78	1.25	1.57
6	10	54	70	1	1.43
7	6	66.5	69	1	1.29
8	12	58	80	1.38	1.86
9	13	65	125	1.8	2.5
10	18	66	110	1.43	2.43
11	23	71	124	1.5	1.9
12	23	77	132	1.88	2.1

**Table 2:** Characteristics of the two subscales: Basic Cognitive Abilities and Communication Abilities.

	Description
***Basic Cognitive Concepts***	
Object Recognition	Identifies familiar objects among distractors based on visual characteristics.
Object Permanence Recognition	Understands that objects continue to exist even when out of sight.
Color Discrimination	Distinguishes and selects objects based on color differences.
Geometric Form Discrimination	Recognizes and differentiates basic geometric shapes.
Cause–Effect Relationship	Understands that specific actions lead to predictable outcomes.
Anticipation of Familiar Events	Anticipates routine events based on contextual or visual cues.
Object-Function Matching	Matches objects with their appropriate use or function.
Quantity Discrimination	Compares sets of objects based on quantity or number.
Object Categorization by Function	Groups objects according to shared functional properties.
Understanding Simple Sequences	Orders steps in simple routines or sequences correctly.
Human Body Discrimination	Recognizes and identifies body parts on self or images.
Spatial Concepts	Understands basic spatial terms (e.g., above, below, or near).
Measure Concepts	Differentiates objects based on size, weight, or length.
Time Concepts	Understands simple temporal relations (e.g., before, after, or now).
***Communication Abilities***	
Respond to Name/Verbal Prompt	Reacts to being called or prompted by turning, looking, or acknowledging.
Express Need—Non-Verbal	Requests items or actions through gestures, gaze, or pointing.
Express Refusal—Non-Verbal	Indicates rejection using body language or facial expression.
Shared Attention (Gaze Alternation)	Alternates gaze between person and object to share focus.
Understanding Gestures from Others	Responds appropriately to familiar social gestures.
Use Context-Based Vocalizations	Produces contextually relevant sounds or vocal patterns.
Participate in Routine Exchanges	Engages in turn-taking or structured social interaction routines.
Express Need—Picture	Uses images to communicate wants or needs.
Match Need to Picture	Identifies images corresponding to presented needs or objects.
Express Need—Written words	Uses written words to communicate wants or needs.
Match Need to Written Word	Identifies written words corresponding to presented needs or objects.

***Note.*** *A complete description of basic cognitive abilities is provided in Appendix A; details regarding communicative abilities are presented in Appendix B.*

**Table 3:** Descriptive statistics for cognitive basic skills across time points by group ^1^.

Skills	Group	Baseline (T0)	3 Months (T1)	6 Months (T2)	9-Month Follow-Up (T3)
Object recognition	Experimental	2.80 (1.48)	3.56 (1.33)	4.80 (0.91) *	4.18 (0.94) *
	Control	2.84 (1.21)	3.22 (1.01)	3.65 (0.88)	3.72 (1.01)
Object permanence recognition	Experimental	1.80 (0.42)	2.89 (1.45)	3.33 (0.94)	3.20 (0.91)
	Control	1.90 (0.43)	3.00 (1.45)	3.34 (0.95)	3.10 (0.88)
Color discrimination	Experimental	1.73 (0.42)	2.33 (1.45)	3.01 (0.94)	3.20 (0.66)
	Control	1.80 (0.42)	2.22 (1.45)	3.33 (0.94)	3.19 (0.91)
Geometric form discrimination	Experimental	1.70 (1.33)	2.00 (1.00)	2.35 (1.25)	2.21 (1.20)
	Control	1.70 (1.33)	2.00 (1.00)	2.35 (1.25)	2.21 (1.20)
Cause–effect relationship	Experimental	1.00 (0.00)	1.30 (0.22)	1.88 (0.66)	1.80 (0.65)
	Control	1.00 (0.00)	1.20 (0.32)	1.77 (0.45)	1.70 (0.33)
Anticipation of familiar events	Experimental	1.00 (0.00)	1.70 (0.27)	2.00 (0.43)	1.99 (0.41)
	Control	1.00 (0.00)	1.63 (0.29)	2.00 (0.36)	1.87 (0.47)
Object-function matching	Experimental	1.60 (1.10)	2.00 (1.00)	2.30 (1.05)	2.30 (1.03)
	Control	1.58 (1.22)	1.99 (0.99)	2.30 (1.05)	2.32 (0.66)
Quantity discrimination	Experimental	1.40 (1.00)	2.10 (0.90)	2.25 (0.95)	2.28 (0.91)
	Control	1.32 (0.89)	1.77 (0.90)	2.00 (0.91)	2.11 (0.83)
Object categorization by function	Experimental	2.20 (0.95)	2.40 (0.77)	2.30 (0.93)	2.05 (0.90)
	Control	2.10 (0.75)	2.34 (0.90)	2.00 (0.91)	2.10 (0.77)
Understanding simple sequences	Experimental	2.30 (1.00)	2.00 (0.95)	2.85 (0.98)	2.98 (0.95)
	Control	2.20 (0.91)	1.80 (0.90)	2.90 (0.93)	2.95 (0.90)
Human body discrimination	Experimental	2.40 (1.07)	1.89 (1.17)	2.15 (1.12)	2.20 (1.10)
	Control	2.33 (1.07)	1.77 (0.89)	2.00 (1.12)	2.10 (0.88)
Spatial concepts	Experimental	1.50 (0.53)	1.83 (0.50)	2.53 (0.51) *	2.45 (0.50) *
	Control	1.50 (0.53)	1.73 (0.67)	1.92 (0.83)	2.00 (0.81)
Measure concepts	Experimental	2.40 (0.97)	2.00 (0.50)	2.20 (0.73)	2.25 (0.75)
	Control	2.40 (0.97)	2.00 (0.50)	2.20 (0.73)	2.25 (0.75)
Time concepts	Experimental	1.30 (0.48)	1.44 (0.53)	1.57 (0.51)	1.40 (0.50)
	Control	1.22 (0.72)	1.50 (0.72)	1.53 (0.51)	1.39 (0.77)

^1^ T-values, *p*-values, effect sizes (Cohen’s *d*), and 95% confidence intervals for comparisons between T0 and T2 are reported in Appendix C due to space constraints. * = *p* < 0.05 for between-group comparison (independent sample *t*-test). Only statistically significant differences are marked.

**Table 4:** Descriptive statistics for communication skills across time points by group ^1^.

Skills/Groups	Baseline (T0)	3 Months (T1)	6 Months (T2)	9-Month Follow-Up (T3)
1. Respond to name/verbal prompt				
Experimental	3.50 (0.80)	3.70 (0.70)	4.20 (0.70)	4.10 (0.60)
Control	3.20 (0.90)	3.30 (0.90)	3.80 (0.80)	3.70 (0.80)
2. Express need—non-verbal				
Experimental	3.80 (0.63)	4.10 (0.57)	4.50 (1.08)	4.20 (1.08)
Control	3.25 (0.89)	3.75 (0.89)	4.17 (0.84)	3.97 (0.84)
3. Express refusal—non-verbal				
Experimental	3.00 (0.90)	3.30 (0.90)	3.60 (0.80)	3.50 (0.80)
Control	2.70 (1.00)	3.10 (1.00)	3.50 (0.90)	3.00 (0.90)
4. Shared attention (gaze alternation)				
Experimental	2.90 (1.00)	3.10 (1.00)	3.80 (0.90)	3.90 (0.90)
Control	2.50 (1.10)	2.70 (1.10)	3.20 (1.00)	3.70 (1.00)
5. Understanding gestures from others				
Experimental	2.30 (0.90)	3.50 (0.80)	3.60 (0.70)	3.70 (0.70)
Control	2.20 (0.90)	3.30 (0.90)	3.40 (0.80)	3.30 (0.80)
6. Use context-based vocalizations				
Experimental	2.10 (1.00)	2.30 (0.90)	2.40 (0.90)	2.45 (0.80)
Control	2.00 (1.00)	2.10 (1.00)	2.30 (0.90)	2.10 (0.90)
7. Participate in routine exchanges				
Experimental	2.20 (0.90)	2.40 (0.80)	2.50 (0.80)	2.60 (0.70)
Control	2.30 (1.00)	2.40 (0.90)	2.50 (0.90)	2.40 (0.90)
8. Express need—picture				
Experimental	1.50 (1.27)	2.20 (1.03)	2.50 (1.18)	2.60 (1.15)
Control	1.78 (1.36)	2.05 (1.39)	2.35 (1.55)	2.40 (1.50)
9. Match need to picture				
Experimental	2.10 (1.10)	2.40 (1.10)	2.80 (1.23)	2.90 (1.20)
Control	1.90 (1.07)	2.13 (1.25)	2.63 (1.30)	2.70 (1.25)

^1^ T-values, *p*-values, effect sizes (Cohen’s *d*), and 95% confidence intervals for comparisons between T0 and T2 are reported in Appendix D due to space constraints.

## Data Availability

Data are available as Supplementary Materials for the present study.

## Supplementary Materials

The following supporting information can be downloaded at https://www.mdpi.com/article/10.3390/children12070928/s1, data file. The supplementary materials includes the full dataset containing all individual-level variables for the participating girls with Rett syndrome, along with their corresponding outcome measures.

## Abbreviations

The following abbreviations are used in this manuscript:

RTT	Rett Syndrome
TR	Telerehabilitation
VR	Virtual Reality
GAIRS	Global Assessment and Intervention Rating Scale
VABSs	Vineland Adaptive Behavior Scales
RARS	Rett Assessment Rating Scale

## Appendix A

**Ability**	**Assessment Procedure**
**Basic Cognitive Abilities**	
Object recognition	The therapist prepares target stimuli with objects of daily use (e.g., a plate, a glass, a phone, or a bottle). The participant sits at a table and has the target on her right along with a distractor on her left. The participant is asked to look at them and to choose the target by looking at it and/or touching it. Then, the position of the stimuli is changed, and the whole procedure is repeated three times with the stimuli in a random order.
Object permanence recognition	The participant is seated at a table facing the therapist. A preferred object or food item is shown, then visibly hidden under a cup or tissue. The participant is encouraged to locate the hidden item by visually tracking it and, if motor abilities permit, by reaching for and retrieving it. The procedure is repeated three times, with the hiding location varied.
Color discrimination	The therapist prepares target stimuli with identical objects of different colors. The participant sits at a table and has the target (e.g., a red cube) on her right and a distractor (e.g., a cube of another color) on her left. She is asked to look at them and to choose the target by looking at it and/or touching it. The position of the stimuli is changed, and the whole procedure is repeated three times with the stimuli in a random order.
Geometric form discrimination	The therapist prepares target stimuli with geometric forms such as a circle, a square, a triangle, a rectangle, or a rhombus. The participant sits at a table and has the target (e.g., a circle) on her right and a distractor (e.g., a triangle) on her left. She is asked to look at them and to choose the target by looking at it and/or touching it. The position of the stimuli is changed, and the whole procedure is repeated three times with the stimuli in a random order.
Cause–effect relationship	The therapist prepares target stimuli with figures depicting a sequence of events: a cause and effect scene. The participant sits at a table and has the target (e.g., a girl falls off the bike) on her right and a distractor (e.g., the girl cries) on her left. She is asked to look at them and to choose the target by looking at it and/or touching it. The position of the stimuli is changed, and the whole procedure is repeated three times with the stimuli in a random order.
Anticipation of familiar events	The therapist selects a familiar and highly routinized activity for the participant (e.g., snack time or going to bed). The activity is always preceded by the same verbal or visual cue (e.g., showing a spoon before snack time). After several repetitions, the therapist presents only the anticipatory cue and asks the participant to select the activity related to it. The trial is repeated three times with different cues associated with distinct events.
Object-function matching	The therapist presents two or more everyday objects (e.g., a toothbrush, a spoon, or a hairbrush) and a corresponding picture or symbolic cue (e.g., an image of teeth, food, or hair). The participant is asked to match the object to its function by selecting or touching the correct object that corresponds to the presented cue. The trial is repeated with at least three different sets of objects and functions, with the position of the items randomized each time.
Quantity discrimination	The therapist prepares visual stimuli showing sets of objects in different quantities (e.g., two apples vs. five apples). The participant is asked to choose the larger or smaller quantity, either verbally or by pointing, touching, or looking at the correct image. The stimuli are presented in pairs, and the position of the larger/smaller group is alternated randomly.
Object categorization by function	The therapist presents an image of a familiar object along with two additional images: one representing the correct function of the object (e.g., eating for a spoon or personal care for a comb) and one unrelated distractor. The participant is asked to identify the correct function of the object by selecting the appropriate image, either through pointing or gaze fixation.
Understanding simple sequences	The therapist uses picture cards or object-based steps of a familiar routine (e.g., putting on socks or brushing teeth). The cards are initially presented in the correct order, and the participant is asked to observe the sequence. Then, the cards are presented in a scrambled order, and the participant is asked to rearrange or indicate the correct order (via gaze, pointing, or verbal cue). The task is repeated with at least two different routines.
Human body discrimination	The therapist prepares target stimuli with body parts. The participant sits at a table and has the target (e.g., belly) on her right and a distractor (e.g., foot) on her left. She is asked to look at them and to choose the target by looking at it and/or touching it. The position of the stimuli is changed, and the whole procedure is repeated three times with the stimuli in a random order.
Spatial concepts	The therapist prepares target stimuli with an object placed in 10 different ways: inside/out, above/below, front/behind, ear/far, and right/left. The participant sits at a table and has the target (e.g., inside) on her right and a distractor (e.g., out) on her left. She is asked to look at them and to choose the target by looking at it and/or touching it. The position of the stimuli is changed, and the whole procedure is repeated three times with the stimuli in a random order.
Measure concepts	The therapist prepares target stimuli with six objects, which are large and tight, high and low, and long and short. The participant sits at a table and has the target (e.g., large) on her right and a distractor (e.g., tight) on her left. She is asked to look at them and to choose the target by looking at it and/or touching it. The position of the stimuli is changed, and the whole procedure is repeated three times with the stimuli in a random order.
Time concepts	The therapist prepares target stimuli with images representing a sequence: before, after, and finally. The participant sits at a table and has the target (e.g., before) on her right and a distractor (e.g., after) on her left. She is asked to look at them and to choose the target by looking at it and/or touching it. The position of the stimuli is changed, and the whole procedure is repeated three times with the stimuli in a random order.
**Communication Abilities**	
Respond to name/verbal prompt	The therapist calls the participant’s name or gives a simple verbal cue (e.g., “Look here”). The participant is expected to respond by orienting her gaze, turning her head, or making another observable acknowledgment. The prompt is repeated several times with variations in intonation and distance to assess consistency.
Express need—non-verbal	In the assessment of this ability, the therapist observes the participant in different daily situations with the collaboration of caregivers and verifies if the participant communicates some basic needs, such as drink, eat, and go to the bathroom, with vocalization or facial expressions.
Express refusal—non-verbal	An undesired or non-preferred item or activity is presented (e.g., an object the participant usually refuses). The participant is encouraged to indicate refusal through non-verbal means such as turning away, pushing the object, shaking the head, or other body language indicating rejection.
Shared attention (gaze alternation)	The therapist presents an interesting object or event and then shifts their gaze between the object and the participant, encouraging the participant to do the same. Alternating their gaze between the object and the therapist is considered an indicator of shared attention. This is repeated across different contexts to assess intentionality.
Understanding gestures from others	The therapist uses simple, commonly understood gestures (e.g., waving goodbye, pointing to an object, or nodding for “yes”). The participant is expected to demonstrate understanding by responding appropriately—e.g., looking toward the pointed object, waving back, or acting accordingly.
Use context-based vocalizations	The participant is observed during structured interactions (e.g., snack time or play) for the use of intentional vocalizations that appear to be linked to the context (e.g., sounds indicating satisfaction, requests, or attention seeking). The therapist notes whether vocalizations are spontaneous and appropriately timed.
Participate in routine exchanges	The therapist engages the participant in familiar social routines (e.g., “peek-a-boo,” “give and take,” or turn-taking games). Participation may include gestures, vocalizations, gaze, or movement that aligns with the expected turn in the routine. Multiple routines are used to assess generalization.
Express need—picture	The participant is presented with pictures of preferred items. When motivated, she is encouraged to select the image corresponding to her need (e.g., water or toy) by pointing, touching, or looking at the picture.
Match need to picture	The therapist presents a real object or situation (e.g., a drink or the start of an activity) and asks the participant to select the corresponding picture among distractors. Correct matching demonstrates an understanding of symbolic representations of needs.
Express need—written word	The participant is presented with written words corresponding to preferred items (e.g., water or toy) alongside distractors. When motivated, she is encouraged to indicate the written word that represents her current need by pointing, touching, or looking at it. A correct selection demonstrates the ability to recognize basic written vocabulary related to personal needs.
Match need to written word	The therapist presents a real object or situation (e.g., offering a snack or initiating an activity) and asks the participant to select the corresponding written word among distractors. Correct matching indicates an understanding of the association between real-world needs and their written linguistic representations.

## Appendix B

*Example of material used for object recognition (e.g., table)*		

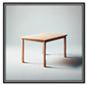

**1st Time**		
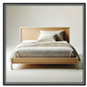
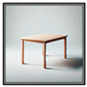
**2nd Time**		
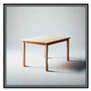
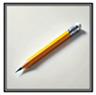
**3rd Time**		
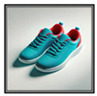
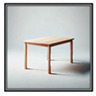

*Example of material used for color discrimination (e.g., red).*		

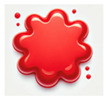

**1st Time**		
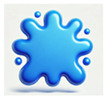
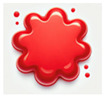
**2nd Time**		
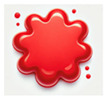
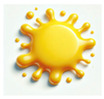
**3rd Time**		
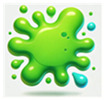
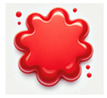

*Example of material used for time discrimination (e.g., before).*		

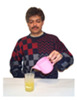

**1st Time**		
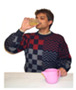
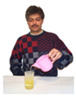
**2nd Time**		
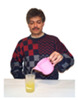
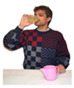
**3rd Time**		
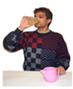
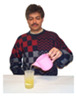

*Example of material used for spatial concepts discrimination (e.g., inside).*		

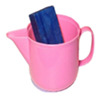

**1st Time**		
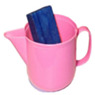
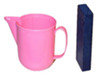
**2nd Time**		
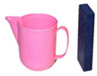
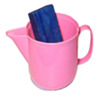
**3rd Time**		
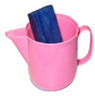
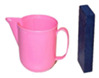

*An example of the cards used for the ability to recognize and express a basic need through pictures*

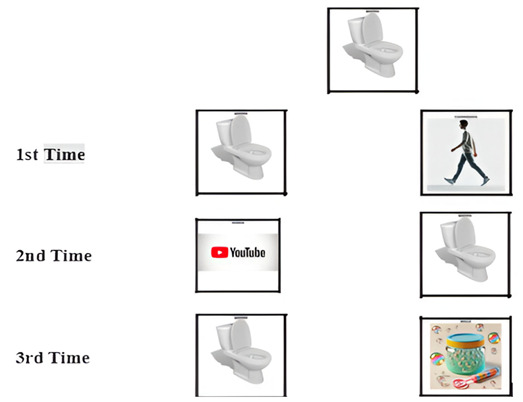

## Appendix C

Paired sample *t*-Tests comparing T0 and T2 scores for each ability and group (complement of Table 3).

**Skills**	**t (Exp)**	***p* (Exp)**	**Cohen’s d (Exp)**	**95% CI (Exp)**	**t (Ctrl)**	***p* (Ctrl)**	**Cohen’s d (Ctrl)**	**95% CI (Ctrl)**
Object Recognition	4.40	<0.001	1.63	[0.98, 2.27]	2.40	0.025	0.77	[0.29, 1.25]
Object Permanence Recognition	5.30	<0.001	2.10	[1.35, 2.86]	4.90	<0.001	1.95	[1.23, 2.67]
Color Discrimination	4.80	<0.001	1.76	[1.08, 2.43]	5.30	<0.001	2.10	[1.35, 2.86]
Geometric Form Discrimination	1.60	0.12	0.50	[0.06, 0.95]	1.60	0.12	0.50	[0.06, 0.95]
Cause–Effect Relationship	4.90	<0.001	1.89	[1.18, 2.59]	5.70	<0.001	2.42	[1.58, 3.26]
Anticipation of Familiar Events	6.80	<0.001	3.29	[2.22, 4.36]	7.90	<0.001	3.93	[2.68, 5.18]
Object-Function Matching	1.90	0.07	0.65	[0.19, 1.12]	1.80	0.08	0.63	[0.17, 1.10]
Quantity Discrimination	2.60	0.016	0.87	[0.38, 1.37]	2.30	0.03	0.76	[0.28, 1.23]
Object Categorization by Function	0.30	0.77	0.11	[−0.32, 0.53]	−0.35	0.73	−0.12	[−0.54, 0.30]
Understanding Simple Sequences	1.70	0.10	0.56	[0.10, 1.01]	2.30	0.03	0.76	[0.28, 1.24]
Human Body Discrimination	−0.70	0.48	−0.23	[−0.66, 0.20]	−0.90	0.38	−0.30	[−0.73, 0.13]
Spatial Concepts	5.40	<0.001	1.98	[1.25, 2.71]	1.70	0.10	0.60	[0.14, 1.06]
Measure Concepts	−0.70	0.48	−0.23	[−0.66, 0.19]	−0.70	0.48	−0.23	[−0.66, 0.19]
Time Concepts	1.70	0.10	0.55	[0.09, 1.00]	1.60	0.12	0.50	[0.05, 0.94]
Compound Means Score on Basic Cognitive Skills	4.11	<0.001	0.84	[0.37, 1.30]	2.92	0.08	0.61	[0.16, 1.06]
Note. Inferential results (from T0 to T2) are reported here for each cognitive ability and group, and complement the descriptive data shown in Table 3 of the main manuscript.								

Paired sample *t*-tests comparing T0 and T2 scores for each ability and group (complement of Table 4).

## Appendix D

**Skill**	**t (Exp)**	***p* (Exp)**	**d (Exp)**	**95% CI (Exp)**	**t (Ctrl)**	***p* (Ctrl)**	**d (Ctrl)**	**95% CI (Ctrl)**
Object Recognition	5.26	<0.001	1.63	[0.98, 2.27]	3.18	0.004	0.77	[0.29, 1.25]
Object Permanence Recognition	6.72	<0.001	2.10	[1.35, 2.86]	6.25	<0.001	1.95	[1.23, 2.67]
Color Discrimination	5.65	<0.001	1.76	[1.08, 2.43]	6.72	<0.001	2.10	[1.35, 2.86]
Geometric Form Discrimination	1.94	0.065	0.50	[0.06, 0.95]	1.94	0.065	0.50	[0.06, 0.95]
Cause–Effect Relationship	6.09	<0.001	1.89	[1.18, 2.59]	7.83	<0.001	2.42	[1.58, 3.26]
Anticipation of Familiar Events	9.72	<0.001	3.29	[2.22, 4.36]	11.60	<0.001	3.93	[2.68, 5.18]
Object–Function Matching	2.70	0.012	0.65	[0.19, 1.12]	2.60	0.015	0.63	[0.17, 1.10]
Quantity Discrimination	3.33	0.003	0.87	[0.38, 1.37]	2.96	0.007	0.76	[0.28, 1.23]
Object Categorization by Function	0.45	0.655	0.11	[−0.32, 0.53]	−0.49	0.627	−0.12	[−0.54, 0.30]
Understanding Simple Sequences	2.29	0.031	0.56	[0.10, 1.01]	2.98	0.007	0.76	[0.28, 1.24]
Human Body Discrimination	−1.05	0.305	−0.23	[−0.66, 0.20]	−1.37	0.184	−0.30	[−0.73, 0.13]
Spatial Concepts	6.31	<0.001	1.98	[1.25, 2.71]	2.55	0.018	0.60	[0.14, 1.06]
Measure Concepts	−1.04	0.309	−0.23	[−0.66, 0.19]	−1.04	0.309	−0.23	[−0.66, 0.19]
Time Concepts	2.26	0.033	0.55	[0.09, 1.00]	2.13	0.044	0.50	[0.05, 0.94]
Compound Means Score on Communication Skills	5.32	<0.001	1.64	[0.96, 2.32]	4.18	<0.001	1.04	[0.47, 1.61]
Note. Inferential results (from T0 to T2) are reported here for each cognitive ability and group, and complement the descriptive data shown in Table 4 of the main manuscript.								
